# One welfare: Linking poverty, equid ownership and equid welfare in the brick kilns of India – ERRATUM

**DOI:** 10.1017/awf.2023.14

**Published:** 2023-02-15

**Authors:** LM Kubasiewicz, T Watson, SL Norris, N Chamberlain, C Nye, RK Perumal, R Saroja, Z Raw, FA Burden

*A non-unique DOI was erroneously assigned to this article at the time of its publication. The article has now been assigned a new DOI. Both the PDF and XML versions have been updated accordingly. The original and new DOIs of this article are provided below.*

*Original DOI:* 10.7120/09627286.31.4.004

*New DOI:* 10.1017/S0962728600032504
